# SASqPCR: Robust and Rapid Analysis of RT-qPCR Data in SAS

**DOI:** 10.1371/journal.pone.0029788

**Published:** 2012-01-06

**Authors:** Daijun Ling

**Affiliations:** Department of Neuroscience, Beckman Research Institute of City of Hope, Duarte, California, United States of America; Friedrich-Loeffler-Institut, Germany

## Abstract

Reverse transcription quantitative real-time PCR (RT-qPCR) is a key method for measurement of relative gene expression. Analysis of RT-qPCR data requires many iterative computations for data normalization and analytical optimization. Currently no computer program for RT-qPCR data analysis is suitable for analytical optimization and user-controllable customization based on data quality, experimental design as well as specific research aims. Here I introduce an all-in-one computer program, SASqPCR, for robust and rapid analysis of RT-qPCR data in SAS. This program has multiple macros for assessment of PCR efficiencies, validation of reference genes, optimization of data normalizers, normalization of confounding variations across samples, and statistical comparison of target gene expression in parallel samples. Users can simply change the macro variables to test various analytical strategies, optimize results and customize the analytical processes. In addition, it is highly automatic and functionally extendable. Thus users are the actual decision-makers controlling RT-qPCR data analyses. SASqPCR and its tutorial are freely available at http://code.google.com/p/sasqpcr/downloads/list.

## Introduction

Quantitative reverse transcription real-time polymerase chain reaction (RT-qPCR) is widely used in biomedical research and diagnostic applications for measurement of relative gene expression. RT-qPCR quantification are easily obscured by non-specific confounding factors resulted from sample-to-sample and run-to-run experimental variations even following standardized experimental methods and data collection criterion [1], [2]. The most important quality control for RT-qPCR quantification is to find an accurate normalizer across samples [3], [4]. Conventional data normalization uses one or few reference genes that are determined before experiments. These genes are commonly used as references for different experiments. Practically, however, no gene has stable expression under various experimental conditions. Emerging evidence have showed that the expression stability of classical reference genes varies greatly with experimental conditions [5], [6], [7], supporting the necessity for data-specific validation of reference genes. A practical strategy for robust data normalization is to measure multiple (≥10) reference genes as reference candidates. Standard statistical algorithms have to be iteratively implemented to determine what particular genes and how many genes should be selected from the reference candidates to achieve a better normalizer for a particular dataset [7], [8]. Currently, however, no efficient and flexible program is available for RT-qPCR data analysis with incorporation of the standard statistical algorithms for data-specific reference validation and analytical optimization.

SASqPCR (Supporting Information file “SASqPCR.sas”), developed using SAS software, is an all-in-one computer program allowing users to perform RT-qPCR data analysis in a more flexible and convenient way. The program provides a dynamic interface for user-controllable customization based on data quality, experimental design as well as specific research aims. Users can easily perform unlimited iterative computations for testing various combinations of different analytical strategies or customized analytical processes. This manuscript briefly describes the working rationale of this program. A real example is provided to illustrate the application of the program for easy, fast and automatic data analysis. The key algorithms, equations and annotated codes for the program have been additionally described in a SASqPCR Tutorial document available at http://code.google.com/p/sasqpcr/downloads/list; however, understanding of this knowledge as well as extensive SAS programming knowledge is not required for general users in application of SASqPCR.

## Methods

### The workflow of SASqPCR

The key RT-qPCR variable used for quantification of gene expression is threshold cycle (Ct). Ct values are primarily determined by the gene-specific cDNA concentrations contributed by particular biomedical conditions but are also confounded by variations in sample preparation. Confounding variations among samples have to be minimized by data normalization using one or multiple internal reference genes [7]. Thus data analysis of raw Ct values includes evaluation of PCR efficiencies, validation of reference genes across samples, normalization of raw Ct values and comparison of gene expression in parallel samples. Some of the computations may be iteratively performed for analytical optimization based on data quality, experimental design and specific research aims.

**Figure 1:**
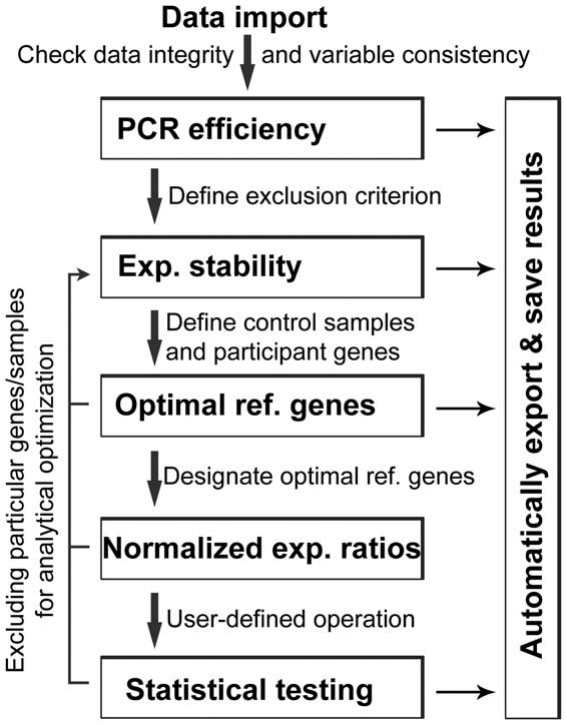
The workflow of SASqPCR. Raw Ct data is imported as a temporary SAS dataset. It is recommended, but not required, to check data integrity and variable consistency using proper SAS procedures. SASqPCR sequentially calculates PCR efficiency, expression stability of candidate reference genes, optimal reference genes, normalized relative expression of target genes, and makes statistical testing. Results are automatically exported and saved. The exported results from each analytical step may serve as a reference for assigning input macro variables for the next step in the workflow. Users can also customize their analyses by arbitrarily excluding particular genes and/or samples. The interface of different computational components allows users to get optimal results. The “user-defined operation” refers to any user-developed programs to extend the analytical function of SASqPCR. Exp., expression; Ref., reference.

SASqPCR contains 5 macros designed for different computational tasks including estimation of PCR efficiencies (*%Efficiency*), evaluation of expression stability of candidate reference genes (*%Stability*), determination of the optimal number of reference genes for robust data normalization (*%Optimization*), calculation of normalized expression ratios of target genes (*%Normalization*), and statistical comparisons of target gene expression between parallel samples (*%Exp_R*). These analytical tasks will be implemented sequentially following a standard analytical workflow (Fig. 1). Each macro has one or more macro variables (Table 1) allowing users to optimize and customize their data analyses. Results from each analytical step are automatically exported and saved in a user-defined Excel file.

**Table 1:** Macro variables of SASqPCR.

Macro Variable	Type	Annotation
*%Efficiency* (dilut, grad);		
dilut	Numerical	Dilution factor of standard cDNA samples
grad	Numerical	Order of dilution series
*%Stability* (E_excluded, References, Count);		
E_excluded	Numerical	Exclusion criterion based on PCR efficiencies
References	Character	List of individual reference genes
Count	Numerical	Number of reference genes
*%Optimization* (Control, Count);		
Control	Character	Name of control cDNA samples
Count	Numerical	Number of reference genes
*%Normalization* (Control, Opt_Ref, N);		
Control	Character	Name of control cDNA samples
Opt_Ref	Character	List of validated reference genes
N	Numerical	Number of optimized reference genes
*%Exp_R* (Target);		
Target	Character	List of target genes

### Estimation of PCR efficiency

Correction of PCR efficiencies is highly recommended in quantification of gene expression using real-time PCR [4]. In quantifiable PCR stage, the concentrations of PCR products increases exponentially (2^n^) with PCR cycles (n). Practically, however, different primer sets may not work equally well. The difference in PCR efficiencies (E) will affect the cycle-dependent concentration of PCR product (C), i.e., 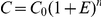, where C_0_ is the initial concentration of the gene-specific cDNA. Mathematically PCR efficiency is the percentage of successful PCR amplification of gene-specific cDNA in each cycle. It is estimated using serially-diluted standard cDNA samples prepared under the same experimental condition as unknown cDNA samples. The macro *%Efficiency* is for calculation of PCR efficiency based on the equation 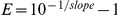, where a linear regression model is fit to the log-transformed relative concentrations of serially diluted standard cDNA samples plotted against their corresponding Ct values.

### Expression stability of reference genes

Gene-specific cDNA concentrations are measured using Ct values. However, the Ct is influenced by both specific biological conditions and confounding variations that are non-specific and non-reproducible in experiments. To offset the confounding variations, stably-expressed internal reference genes are measured simultaneously along with target genes for data normalization across parallel samples. Internal reference genes are usually selected from classical housekeeping genes that are mostly recognized based on their biological functions and frequently used as general-purpose reference genes in different experiments. However, there is no absolute “housekeeping” gene with respect to the stability of mRNA transcription, thus precluding the suitability using the universal reference genes for various experimental conditions. Therefore internal reference genes for robust data normalization have to be validated for their expression stability across samples for each experiment [5], [7].

The macro *%Stability* evaluates expression stability of candidate reference genes. The method is developed primarily based on the algorithm of geNorm, the most widely-used program for validation of reference genes [4], [9], [10]. The key variable indexing the relative expression stability of reference genes is M value, calculated as the mean standard deviation of the log-transformed expression ratios across samples for a particular gene relative to other genes remaining in the gene panel of reference candidates [7], [9]. The calculation is iteratively performed by stepwise exclusion of individual genes with the highest M value (i.e. the least stable gene) from the panel until reaching the last three genes. The most stable gene with the smallest M value is identified as a single gene in SASqPCR rather than two genes tied for the best stability. In addition, the expression stability of candidate genes is ranked based on their reverse order of stepwise exclusion in iterative calculation cycles rather than the absolute M values as using in the geNorm program [7]. These modifications allow SASqPCR to be more accurate in assessment of the relative expression stability of candidate genes. As a parameter reflecting not only candidate reference genes (across samples) but also cDNA samples (across genes), the rank of expression stability or M values should not be borrowed from one analysis or experiment to another. However, current RT-qPCR users do not notice this important point.

### Normalizer optimization

Inappropriate selection of internal reference genes for data normalization may obscure the actual biological differences among samples. Robust data normalization requires a data-tailored normalizer [7]. To obtain such a normalizer, users need to determine what particular genes and how many genes are sufficient for robust data normalization. The optimal number of reference genes is predicted by the pairwise variation (V_n/n+1_), an index of the relative stability of serial normalization factors (NF) using an increasing number of reference genes [7], [9].

The macro *%Optimization* calculates V_n/n+1_ based on the geNorm algorithm with slight modification. The V_n/n+1_ across samples are the variances of serial log-transformed NF ratios using N relative to N+1 reference genes. Note that the order for stepwise inclusion of individual reference genes is based on their ranked expression stability rather than the magnitudes of their M values. The minimal V_n/n+1_ indicates the most stable NF achievable within a particular set of cDNA samples and a particular panel of reference candidates, thus corresponding to the optimal number of validated reference genes [7]. Similar to the M value, the optimized set of reference genes should not be adopted from one analysis to another due to its relying on particular data.

Compared to other programs, SASqPCR calculates V_n/n+1_ starting from V_1/2_ rather than the usual V_3/4_. This improvement has practical significance for robust data normalization, since it preserves the possibility that single or two properly validated reference genes in some cases are sufficient to normalize target gene expression rather than the necessity of multiple (≥3) genes [7].

### Data normalization

Relative expression of target genes is determined using the macro *%Normalization*. The underlying algorithm is the “delta delta Ct (ΔΔCt)” method [4] by which target gene expression in treated cDNA samples is first compared to control cDNA samples and then normalized to the expression of reference gene(s). The normalizer is calculated as the geometric mean of multiple reference genes [9] or single gene validated in the previous analytical steps. Reference gene(s) for normalizer calculation can also be arbitrarily selected by users. The differential expression of target genes in parallel samples is reported as the fold change to reflect up- or down-regulated expression in response to particular experimental conditions. After running of this macro, a temporary SAS dataset (called “Exp_R”) is generated that can be manipulated for user-defined analytical goals, for example, cluster analysis of expression profiles for multiple genes and samples.

### Normalized expression ratios and statistical test

Normalized expression ratios (R) of target genes and their standard errors (SE) are reported using the macro *%Exp_R*. The calculation of R and SE follows standard algorithms [4], [9]. This macro also performs Student's t-test as a default statistical method for comparison of target gene expression between parallel treatment types. Two-tailed P value is reported. For examining multiple genes/treatment types, users may consider reporting P values sequentially adjusted using Benjamini and Hochberg False Discovery Rate to correct for multiple comparisons [11]. Even though the t-test is the most widely used method for statistical validation of significance in quantitative differences, selection of the appropriate statistical method must be based on particular experimental design and sample size. SASqPCR preserves the capacity for implementation of other statistical methods, for example ANOVA, permutation-based testing, Mann-Whitney-Wilcoxon testing, etc for advanced users.

### Test data

No specific data format is required for application of SASqPCR. Ct values can be saved in an Excel file, a user favorite database file or even as a plain text file. There is no limitation on the size of the raw dataset. At least 5 variables are required for using the program: gene, sample, type, serial and Ct (see SASqPCR Tutorial for the details). Here a real RT-qPCR dataset is used to illustrate how to use SASqPCR. The dataset contains 262 standard and 500 unknown individual real-time PCR reactions. There are 19 candidate reference genes and 9 target genes measured across 3 treatment types relevant to neurodegenerative insults in a *Drosophila* model of Alzheimer's disease [8], [12]. The dataset is sampled from the data using in a previous study [7] where the methods and materials for RT-qPCR experiments have been described. The Ct values of this example data are saved in an Excel spreadsheet “Raw_Ct” in the file “PCR_data.xls” available at http://code.google.com/p/sasqpcr/downloads/list.

## Results

After starting SAS software (SAS Institute Inc., Cary, NC, USA), input and submit the SAS codes shown in Table 2. Users need to arbitrarily designate an Excel file (Code #1 in Table 2) to “myresult” for saving output results. If the designated Excel file does not exist, the program will generate it with saved results into multiple spreadsheets corresponding to the implementation of different analytical steps. If the designated file already exists, the program will automatically update it. Here all results generated from the example data testing are saved in the Excel file “PCR_data.xls” with different spreadsheets as indicated below. The PCR_data.xls is available at http://code.google.com/p/sasqpcr/downloads/list.

**Table 2:** The analytic procedure of the example RT-qPCR data using SASqPCR.

Code#	SAS code for analysis of study example data*
1	Filename myresult “X:\qPCR\PCR_data.xls”; run;
2	proc import out = work.raw_ct datafile = “X:\qPCR\PCR_data.xls”dbms = Excel replace; sheet = “Raw_Ct”; run;
3	*%Include* “X:\qPCR\SASqPCR.sas”; run;
4	*%Efficiency*(10, 5);
5	*%stability*(0.95, “14-3-3e” “Act5C” “Appl” “CG13220” “Cyp1” “Ef1a48D” “Elav”“Exba” “Gapdh2” “GstD1” “Rap2l” “Robl” “RpL13A” “RpL32”“Sdha” “Su(Tpl)” “aTub84B” “eIF-1A” “l(3)02640”, 19);
6	*%Optimization*(“C05”, 19);
7	*%Normalization*(“C05”, “Exba” “Cyp1” “l(3)02640” “Appl” “14-3-3e”, 5);
8	*%Exp_R*(“Atg1” “Aß42” “CathD” “Hsp70” “InR” “Lamp1” “Rab5” “Tor” “Hu_tau”);

*The folder “X:\qPCR” in code #1, #2 and #3 needs to be changed to the appropriate path and filename so that SAS software can successfully access it. Input names of genes and samples must exactly match those in the original dataset. Please note that it is possible but not necessary to use the same Excel file to save the raw Ct data and exported results.

The raw Ct data is imported as a temporary SAS dataset by running Code #2 in Table 2. Users can review the new dataset in the SAS library or check the data integrity and variable consistency using proper SAS codes (not included). Load SASqPCR by running Code #3 in Table 2, given that the program is saved as “X:\qPCR\SASqPCR.sas”. I recommend that the 5 macros be performed sequentially and separately. Users need to input numeric or character values for macro variables as defined in Table 1.

First, estimate PCR efficiencies for all primer sets by running Code #4 in Table 2. Here the standard cDNA samples have 5 step 10 fold dilution series. Output results are automatically saved in a spreadsheet “Efficiency” in the pre-defined Excel file. The results contain multiple parameters including PCR efficiency and squared R for evaluating RT-qPCR quality. Users may exclude particular genes from further analyses based on PCR efficiency, R square or other user-justifiable criteria.

Second, assess the expression stability of candidate reference genes by running Code #5 in Table 2. Users can arbitrarily include or exclude particular genes from analyses by changing the macro variables. Here those genes with PCR efficiency <95% are excluded. Total 19 candidate reference genes participate in the assessment of their relative expression stability. Output results are automatically saved in a spreadsheet “M_value” in the pre-defined Excel file. The results contain M values of the candidate reference genes and their ranked expression stability. Note that the rank sometimes may not exactly match the sorting order of M values, because the rank represents the reverse order of stepwise exclusion of individual genes in each iterative calculation cycle. The priority in selection of particular reference genes for data normalization is based on their rank rather than their M values.

Third, evaluate pairwise V_n/n+1_ to determine the optimal number of reference genes by running Code #6 in Table 2. Here the control cDNA samples are “C05”. Total 19 candidate reference genes participate in the calculation. Output results, containing V_1/2_ to V_18/19_, are automatically saved in a spreadsheet “Pairwise_V” in the pre-defined Excel file. Visualization of the result indicates that the V_5/6_ has the lowest value among the 18 pairwise V_n/n+1_ values (Fig. S1), suggesting that the optimal number of reference genes is 5. Thus 5 reference genes are selected for data normalization from the spreadsheet “M_value” starting from the top: i.e. “*Exba*” “*Cyp1*” “*l(3)02640*” “*Appl*” and “*14-3-3e*” (see “PCR_data.xls” for the detail).

Forth, calculate the normalized expression ratios of target genes and make statistical comparisons among parallel samples by running Code #7 and #8 in Table 2. Here the control cDNA samples are “C05”. The 5 validated reference genes are used for data normalization. The results, saved in a spreadsheet “Exp_ratio” in the pre-defined Excel file, include the normalized expression ratios (R) of target genes between the control and treatment samples, standard errors (SE_R), two-tailed P values by Student's t test, and the P values adjusted for multiple comparisons. The results are visualized using independent software after data analysis (Fig. S2).

It is convenient for users to test different analytical combinations by excluding particular samples and/or genes and iteratively performing the *%Stability*, *%Optimization* and *%Normalization* to optimize results. Thus users can assign particular parameters for each macro variable in each analytical step based on their experimental design, data quality and analysis optimization.

## Discussion

Analysis of RT-qPCR data requires many complex iterative calculations particularly for data-specific validation and optimization of data normalizers. It is thus important for general users to have a simple computer program for both automatic data analyses and user-controllable customization based on particular experimental considerations. SASqPCR is developed particularly for these analytical tasks. It incorporates all computational functions important for RT-qPCR data analysis including assessment of PCR efficiencies, validation of candidate reference genes, normalizer optimization, normalization of confounding variations across samples, and statistical testing of the association of target gene expression with specific experimental conditions. Technically SASqPCR improves the geNorm program in evaluation of the expression stability of candidate reference genes. It also preserves the possibility that an accurate normalizer sufficient for data normalization may be a single, two or multiple properly validated reference genes [7].

SASqPCR emphasizes flexibility and straightforwardness in data analysis. First, the input data is raw Ct values of individual genes and cDNA samples with no limitation to the data size or format. Thus multiple combined datasets or PCR array data can be analyzed. Users can manage and analyze their data in a traditional way rather than relying on the proprietary instruments or plate-based data formats. Users can also include more experimental variables in their datasets and combine the SASqPCR program with any other SAS procedures for their particular analytic purposes. Second, SASqPCR provides a dynamic interface for users to optimize results based on data quality, experimental designs and research aims. By simply changing the key macro variables, users can quickly make numerous iterative computations and thus test various combinations of different analytical strategies. Third, final results exported by SASqPCR are basically numeric values that give users great freedom to determine how to visualize their results for publications or presentations. In addition, users can also check data quality, view intermediate details of the analytical procedures, customize the analytical processes and even extend analyses to use advanced statistical tools. Thus users are the final decision-makers directing the specific types of analytical procedures for their data.

## Supporting Information

Figure S1 **Pairwise variation (V_n/n+1_) of candidate reference genes.** The arrow points to the minimal value (V_5/6_) among 18 pairwise V_n/n+1_ values.
(TIF)

Figure S2 **Normalized expression ratios of target genes.** Target genes: *Atg1* (Autophagy-specific gene 1), *CathD* (Cathepsin D), *Hsp70* (Heat shock protein 70), *InR* (Insulin-like receptor), *Lamp1* (Lysosome associated membrane protein 1), *Rab5* (Rab-protein 5), *Tor* (Target of rapamycin), transgene *Hu_tau* (human microtubule-associated protein tau) and transgene *Aβ42* (human amyloid beta 1–42 peptide). Treatment types: A05 (transgenic animals expressing human Aβ42), T05 (transgenic animals expressing human tau), and C05 (Aβ42/tau noncarriers). The transgenes (*Hu_tau* and *Aβ42*) have high expression ratios due to no expression in the control (C05). For endogenous genes, *Hsp70* expression increases about 6 times in response to the expression of *Hu_tau* but not *Aβ42* transgene. Other target genes have no obvious change in response to the expression of either *Hu_tau* or *Aβ42* transgene.
(TIF)
