# Supplementary figures and images for: SASqPCR: Robust and Rapid Analysis of RT-qPCR Data in SAS
Source: PLoS One. 2012 Jan 6;7(1):e29788. doi: 10.1371/journal.pone.0029788 (PMC3253109; doi:10.1371/journal.pone.0029788)

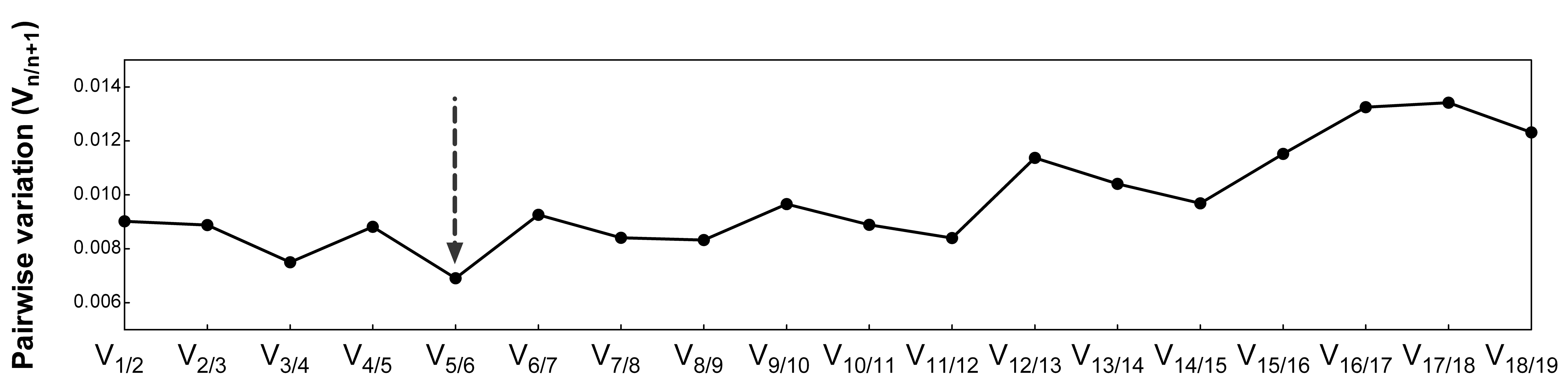

Supplement: Figure S1 — Pairwise variation (Vn/n+1) of candidate reference genes. The arrow points to the minimal value (V5/6) among 18 pairwise Vn/n+1 values. (TIF) [file pone.0029788.s001.tif]

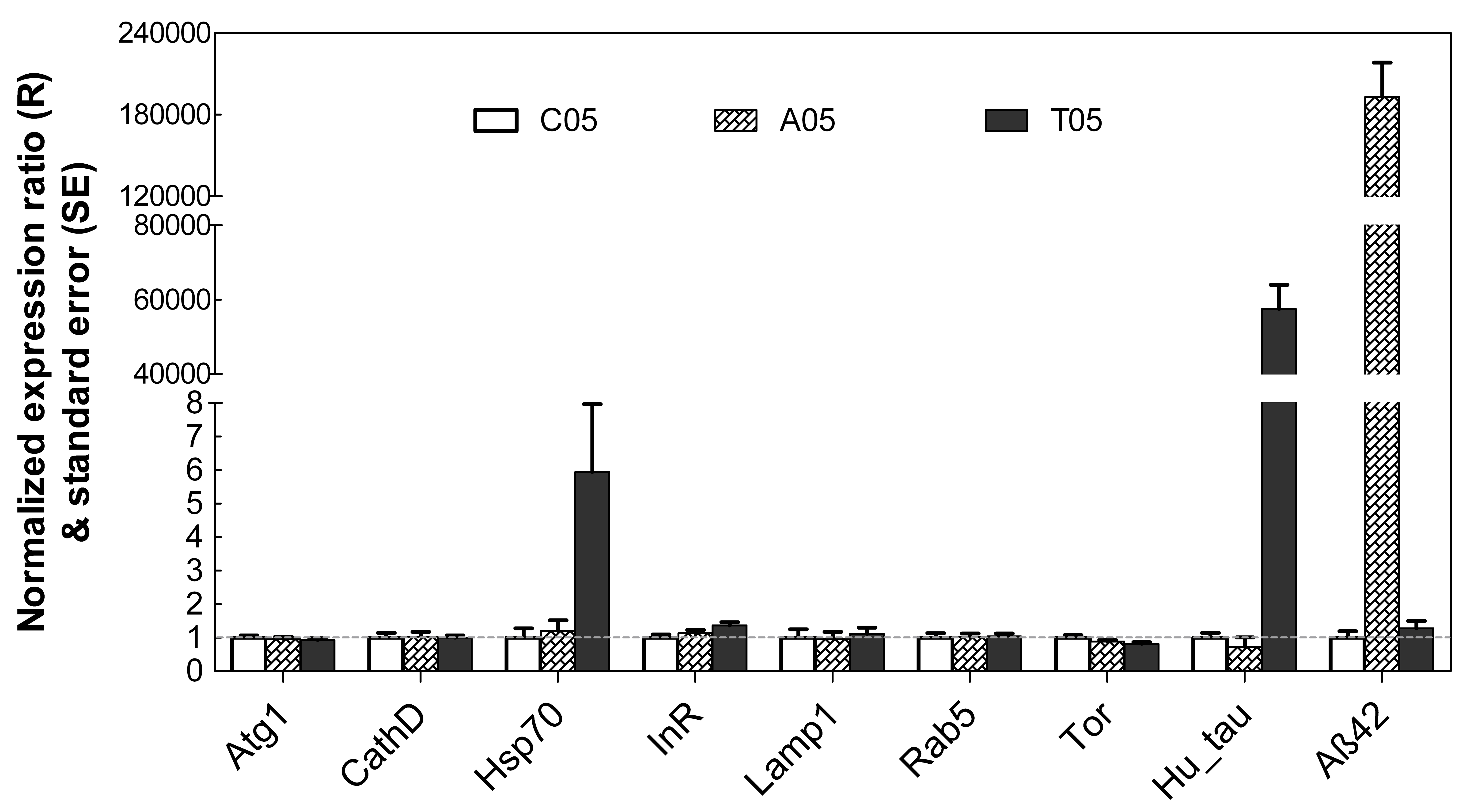

Supplement: Figure S2 — Normalized expression ratios of target genes. Target genes: Atg1 (Autophagy-specific gene 1), CathD (Cathepsin D), Hsp70 (Heat shock protein 70), InR (Insulin-like receptor), Lamp1 (Lysosome associated membrane protein 1), Rab5 (Rab-protein 5), Tor (Target of rapamycin), transgene Hu_tau (human microtubule-associated protein tau) and transgene Aβ42 (human amyloid beta 1–42 peptide). Treatment types: A05 (transgenic animals expressing human Aβ42), T05 (transgenic animals expressing human tau), and C05 (Aβ42/tau noncarriers). The transgenes (Hu_tau and Aβ42) have high expression ratios due to no expression in the control (C05). For endogenous genes, Hsp70 expression increases about 6 times in response to the expression of Hu_tau but not Aβ42 transgene. Other target genes have no obvious change in response to the expression of either Hu_tau or Aβ42 transgene. (TIF) [file pone.0029788.s002.tif]
